# A Designed “Nested” Dimer of Cyanovirin-N Increases Antiviral Activity

**DOI:** 10.3390/v8060158

**Published:** 2016-06-06

**Authors:** Brian W. Woodrum, Jason Maxwell, Denysia M. Allen, Jennifer Wilson, Lauren R.H. Krumpe, Andrey A. Bobkov, R. Blake Hill, Karen V. Kibler, Barry R. O’Keefe, Giovanna Ghirlanda

**Affiliations:** 1 School of Molecular Sciences, Arizona State University, Tempe, AZ 85287-1604, USA; bwoodrum@asu.edu (B.W.W.); jdmaxwe1@asu.edu (J.M.); Denysia.Allen@gmail.com (D.M.A.); 2 Molecular Targets Laboratory, Center for Cancer Research, National Cancer Institute at Frederick, Frederick, MD 21702, USA; wilsonje@mail.nih.gov (J.W.); okeefeba@mail.nih.gov (B.R.O.); 3 Basic Science Program, Leidos Biomedical Research, Inc., Frederick National Laboratory, Frederick, MD 21702, USA; haughl@mail.nih.gov; 4 Sanford Burnham Prebys Medical Discovery Institute, 10901 North Torrey Pines Road, La Jolla, CA 92037, USA; abobkov@sbpdiscovery.org; 5 Department of Biochemistry, Medical College of Wisconsin, Milwaukee, WI 53226, USA; rbhill@mcw.edu; 6 School of Life Sciences and The Biodesign Institute, Arizona State University, Tempe, AZ 85287, USA; Karen.Kibler@asu.edu

**Keywords:** Cyanovirin-N, antiviral lectins, glycan-binding proteins, oligomannose, gp120

## Abstract

Cyanovirin-N (CV-N) is an antiviral lectin with potent activity against enveloped viruses, including HIV. The mechanism of action involves high affinity binding to mannose-rich glycans that decorate the surface of enveloped viruses. In the case of HIV, antiviral activity of CV-N is postulated to require multivalent interactions with envelope protein gp120, achieved through a pseudo-repeat of sequence that adopts two near-identical glycan-binding sites, and possibly involves a 3D-domain-swapped dimeric form of CV-N. Here, we present a covalent dimer of CV-N that increases the number of active glycan-binding sites, and we characterize its ability to recognize four glycans in solution. A CV-N variant was designed in which two native repeats were separated by the “nested” covalent insertion of two additional repeats of CV-N, resulting in four possible glycan-binding sites. The resulting Nested CV-N folds into a wild-type-like structure as assessed by circular dichroism and NMR spectroscopy, and displays high thermal stability with a *T*_m_ of 59 °C, identical to WT. All four glycan-binding domains encompassed by the sequence are functional as demonstrated by isothermal titration calorimetry, which revealed two sets of binding events to dimannose with dissociation constants *K*_d_ of 25 μM and 900 μM, assigned to domains B and B’ and domains A and A’ respectively. Nested CV-N displays a slight increase in activity when compared to WT CV-N in both an anti-HIV cellular assay and a fusion assay. This construct conserves the original binding specifityies of domain A and B, thus indicating correct fold of the two CV-N repeats. Thus, rational design can be used to increase multivalency in antiviral lectins in a controlled manner.

## 1. Introduction

Cyanovirin-N (CV-N) is a small (11 kDa) cyanobacterial protein with potent antiviral activity towards HIV, Ebola, influenza, hepatitis C, and other enveloped viruses [1,2,3,4,5]. Its mechanism of action involves high affinity binding to the oligomannose glycans tethered to the viral envelope glycoproteins, gp120 in the case of HIV. The CV-N solution structure [1,2,3,4,5,6] shows an unique beta sheet fold that comprises two quasi-symmetric glycan binding sites, A (residues 1–38/90–101) and B (residues 39–89), connected on each side by a short helical linker. Each carbohydrate binding site binds Man (1 → 2)Man termini in oligomannose selectively, although domain B binds dimannose more tightly than domain A, and domain A shows selectivity for linear trimannose [1,2,3,4,5,7]. Computational and mutational studies have clarified the contribution of sequence to specificity in the two sites [6,8,9,10,11,12,13]. Site B consists of a contiguous stretch of amino acids, whereas N- and C-terminal residues fold to form glycan binding site A. This unique configuration results in possible dimerization by 3D domain swapping of the CV-N fold, which occurs under some conditions through the extension of a hinge region (residues 50–53), resulting in the creation of four carbohydrate-binding sites A, A´ and B, B´ that retain glycan selectivity [14,15,16,17,18,19]. It has been shown that the 3D domain swapped dimer is a kinetically trapped folding intermediate. Conversion of the domain-swapped dimeric form to the thermodynamically stable monomer is slow in physiological conditions [18,19]. However, interconversion is fast in the conditions used to assay antiviral activity; thus, an accurate comparison of the activity of these forms has been challenging.

The role of multivalency, e.g., the presence of at least two binding domains, for the antiviral activity of CV-N is well established; however, it remains unclear whether activity can be enhanced beyond that of WT by further increasing the number of binding domains [16,19,20,21,22,23,24]. Mutations resulting in inactivation of either site A (P51G-m4-CV-N) [19] or site B (CV-N^mutDB^) [24] cause a significant decrease in binding affinity for gp120 by ELISA and completely eliminate antiviral activity. The crucial role of multivalency for WT activity was demonstrated by the restoration of antiviral activity upon forced dimerization of these defective variants, either through mutations that induced 3D domain swapping [21] or through engineered disulfide linkages [25]. These results show that at least two binding sites are necessary for antiviral activity, regardless of the identity of the domains [26]. Two approaches have been taken to probe whether the activity of WT CV-N could be further enhanced by engineering constructs with more than two binding domains, yielding contradictory results. Stable domain-swapped forms of CV-N obtained through hinge region mutations (ΔQ50)CV-N and (S52P)CV-N display antiviral activity identical to that of WT CV-N, despite comprising four glycan binding domains instead of two [18]. However, a series of covalent dimers of CV-N show 3- to 18-fold enhancement of antiviral activity over the monomeric WT CV-N. These proteins were designed to stabilize the 3D domain swapped dimer by connecting two CV-N sequences through polypeptide linkers of varying length, and fold into a rigid structure resembling the 3D domain-swapped dimer as verified by X-ray crystallography [22]. Surprisingly, further increasing the number of glycan binding sites by linking more than two CV-N sequences in tandem did not increase antiviral activity. The rigid domain swapped arrangement of the CV-N repeats, though, raises the question of whether the enhancement of activity is limited by steric issues that may prevent simultaneous engagement of more than two sites, due to geometric constraints in binding oligomannose displayed on the viral surface [22].

In this work, we introduce a novel covalent dimer as platform to address multivalency and geometric requirements. The design is inspired by natural CV-N homologous proteins, and contains a flexible dimer of CV-N meant to avoid steric constraints and rigidity intrinsic to 3D domain swapping could confer enhanced antiviral activity. To accomplish this, we engineered a novel dimeric CV-N construct, dubbed Nested CV-N, which is built by splicing a second CV-N sequence within the two repeats of a first one. We show that Nested CV-N forms a stable, well folded dimer as designed, with secondary structure content and thermal stability comparable to WT CV-N, and no evidence of oligomer formation. Further, Nested CV-N possesses enhanced binding affinity to gp120 and activity against HIV slightly increased compared to WT. These results support our hypothesis that enhanced antiviral activity derives from multivalency, and that flexible constructs may allow optimal orientation of carbohydrate binding domains on the target gp120 glycoprotein.

## 2. Materials and Methods

### 2.1. Protein Expression and Purification

A synthetic gene encoding for Nested CV-N and containing a pelB leader sequence at the start and a His-tag sequence at the C terminus (GenScript, Piscataway, NJ, USA) was double digested with NdeI and XhoI, and ligated into pET26b(+) vectors using established protocols. The vectors were transformed into NEB5α cells and BL-21(DE3) cells (New England Biolabs, Inc., Ipswich, MA, USA). Protein expression was carried out as previously described [19,27].

The proteins were obtained from the periplasmic fraction, and purified by nickel affinity chromatography (Ni Sepharose High Performance medium, GE Healthcare, Marlborough, MA, USA), using 400 mM imidazole under native conditions for the elution step. Molecular mass and purity were confirmed by Tricine SDS-PAGE gel and by MALDI-TOF mass spectrometry. Amino acid analysis was used to determine extinction coefficients at 280 nm (assay performed at NCI). Protein concentrations were determined by measuring tryptophan absorbance at 280 nm using the experimentally verified extinction coefficient of 19,540 M^−1^ cm^−1^.

### 2.2. Circular Dichroism Spectroscopy (CD)

Circular Dichroism (CD) measurements were performed using a Jasco J-815 spectropolarimeter equipped with a thermostatic cell holder, PTC 424S. Spectra were measured from 240 to 190 nm, using a scanning speed of 50 nm/min and a data pitch of 1.0 nm at 15 °C [28]. Samples contained approximately 15 µM of protein in 10 mM HEPES, pH = 8.0 buffer. Thermal denaturation was monitored on full spectra collected every 2 °C in the 4 to 90 °C temperature range. Detailed thermodynamic analysis was precluded due to the irreversible unfolding of Nested CV-N, which reflects the behavior of WT CV-N [29]. Apparent T_m_ was determined by analyzing the sigmoidal curves recorded at 235 nm using commercially available software (Prism 6, GraphPad Software).

### 2.3. Chemical Denaturation Analysis

Chemical denaturation titrations were performed on pre-equilibrated samples due to the slow folding kinetics of CV-N and its mutants [29]. For each concentration of GdnHCl, a sample containing 10 M protein in 20 mM NaPO_4_ buffer, pH 7.5 was incubated at room temperature for 72 h. The concentration of GdnHCl in each sample was verified by refractometry. Equilibrium denaturation curves were determined both by monitoring the circular dichroism signal as described above, and by monitoring the intrinsic tryptophan fluorescence; the excitation wavelength was set to 290 nm, and fluorescence emission was collected from 300 to 400 nm. Denaturation curves were obtained by plotting I330/I360 tryptophan fluorescence ratio for each sample *versus* Gdn·HCl concentration. The I330/I360 was obtained for each point by exciting at 290 nm and recording emission at 330 and 360 nm for 1 min [29]. The apparent midpoint of the transition for each curve was determined using commercially available software (Prism 6, GraphPad Software, San Diego, CA, USA).

### 2.4. Isothermal Titration Calorimery

Isothermal titration calorimetry (ITC) was performed at the Sanford-Burnham Medical Research Institute Protein Analysis Facility using a ITC200 calorimeter from Microcal (North Hampton, MA, USA). The data were analyzed using Origin software package provided by Microcal. Two separate conditions were used to measure binding of dimannose to Nested CV-N. First, 3.6 mM dimannose was titrated into 100 µM Nested CV-N in 20 mM HEPES for high affinity binding site determination (data not shown). This data were fitted to “one set of sites” model since contribution from low affinity sites was relatively small at these conditions. This measurement experimentally confirmed presence of 2 high-affinity binding sites for dimannose in Nested CV-N. To characterize the low affinity binding sites we have titrated 20 mM dimannose into 100 µM Nested CV-N. The data were fitted to “two set of sites” model to accommodate both low and high affinity binding sites. Thermodynamic values obtained for the high affinity binding sites from the 3.6 mM dimannose titration were used to initialize parameters for the fitting of 20 mM dimanose data. Due to low C value for the low affinity sites, we were not able to determine stoichiometry for the low affinity binding experimentally. However, ITC data are consistent with 2 high/2 low affinity sites per Nested CV-N. To characterize binding of Man9, 1 or 2 mM Man9 was titrated into 20 and 30 µM Nested CV-N, respectively, in 20 mM HEPES. Only one set of sites (4 sites having the same high affinity) was detected for Man9.

### 2.5. Nuclear Magnetic Resonance

^1^H-^15^N HSQC spectra were recorded on a 150 µM Nested CV-N sample uniformly enriched in ^15^N at 14.1T on a Bruker Avance III 600 spectrometer equipped with a Prodigy triple resonance probe, at 312 K with 256 scans per increment, 640 (t2) × 70 (t1) complex points with acquisition times of 76 ms (^1^H) and 63 ms (^15^N). NMR data processing was carried out using NMRPipe [30] and subsequently analyzed by visual inspection compared to wild type CV-N chemical shift assignments using NMRViewJ [31,32].

### 2.6. Binding to gp120

Binding of Nested CV-N to glycosylated soluble gp120 was assessed by enzyme-linked immunosorbent assays (ELISA), using WT CV-N as internal reference [33,34]. Each well of a 96-well plate (Nunc; Maxiscorp, Gaithersburg, MD, USA) was incubated with 100 ng of gp120 (rgp 120 HIV-1 MN, baculovirus; ImmunoDiagnostics, Inc. Woburn, MA, USA) or ribonuclease B in phosphate buffered saline (PBS) for two hours at room temperature. The plates were then rinsed with PBS containing 0.1% Tween 20 (PBST) three times, and blocked by treatment with 3% bovine serum albumin (BSA) at 4 °C overnight. The plates were then washed three times with PBST. Half log serial dilutions of Nested CV-N and WT CV-N were then added to the wells in triplicate, for final concentrations ranging from 0.005 nM to 0.05 µM. The last column was left as a blank, and only contained the BSA. The plates were incubated for one hour and washed three times with PBST. Bound proteins were detected with His-tag targeting reagents. The plates were incubated with 100 μL/well of the HisProbe-HRP (1 μg/mL in PBS-T) (Thermo Scientific, Waltham, MA USA) for 1 h at room temperature. After the plates were washed three times with PBS-T, 100 μL/well of tetramethylbenzidine peroxidase substrate solution (KPL) was added and incubated ~2 min. The reaction was quenched with 100 μL of 2 M H_2_SO_4_ and the absorbance at 450 nm was read.

### 2.7. Antiviral Assays

Cellular viability assay. The anti-HIV activity of Nested CV-N and Nested-P CV-N were assessed in the Molecular Targets Laboratory, NCI at Frederick using a modified XTT assay that measures the cytopathic effect of HIV-1 on CEM-SS cells. Briefly, uninfected CEM-SS cells were plated at a density of 1 × 10^−5^ cells/mL in 50 µL of complete medium in a 96-well plate. Serial dilutions of 100 µL of CV-N as standard and Nested CV-N were added to the wells. The cells were then infected with 50 µL of HIV-1_RF_ virus. The plates were incubated at 37 °C in an atmosphere containing 5% CO_2_ for 6 days. To estimate cellular viability, 50 µL XTT solution was added to each well and incubated for 4 h at 37 °C. Cells that had not been killed by the HIV virus could reduce XTT to a soluble, colored formazan while HIV-infected and subsequently lysed cells could not. Metabolic reduction of XTT to formazan was recorded by absorbance at 450 nm to determine cellular viability. CV-N and variant samples were thawed to room temperature, mixed, and diluted into PBS pH 7.4 at the specified submitted concentration. A total of 2, 60 µL aliquots were submitted for each sample to obtain quadruplicate data points. The samples were serially-diluted using a 10^0.5^ dilution scheme, and HIV induced cytopathicity evaluation was conducted as described [35].

### 2.8. Cell Fusion Assay

BSC-40 cells (ATCC) and TZM-bl cells (NIH AIDS Research and Reference Reagent Program Division of AIDS, NIAID, NIH: from John C. Kappes, Xiaoyun Wu and Tranzyme Inc., Durham, NC, USA) [36,37] were seeded in flasks [both in DMEM (Cell Gro, #10017CV), supplemented with 10% FBS (Fisher-Scientific, Waltham, Massachusetts, USA) for TZM-bl and 5% FBS for BSC-40 cells] and infected when confluency was reached. BSC-40 cells were infected with vTF7-3 (obtained through the NIH AIDS Reagent Program, Division of AIDS, NIAID, NIH: from Tom Fuerst and Bernard Moss) [38] and vCB41 (obtained through the NIH AIDS Reagent Program, Division of AIDS, NIAID, NIH: from Christopher C. Broder, Paul E. Kennedy, and Edward A. Berger), each at an MOI of 3. TZM-bl cells were infected with vCB21R-LacZ (obtained through the NIH AIDS Reagent Program, Division of AIDS, NIAID, NIH: from Christopher C. Broder, Paul E. Kennedy, and Edward A. Berger) [39] virus at an MOI of 3. Infected cells were incubated for 18 h at 32 °C, then were disrupted with Cell Dissociation Buffer (Gibco, Billings, MT, USA). DMEM supplemented with 2.5% FBS was used to dilute proteins, and 160 µL of each dilution was transferred to a well of a 96-well plate. Each cell line, at 4 × 10^4^ cells, was added to the wells containing inhibitor. Controls were wells with only one cell line and no protein, wells with both cell types but no protein, and wells with medium only (no cells or protein). The plate was then incubated at 37 °C for 2.5 h, and then frozen at −80°. On the day of the assay, the plate was thawed, and 10 µL of 10% NP40 was added to each well and mixed by pipetting. The plate was incubated at room temperature for 20 min. Fifty microliters of each sample was transferred to a fresh plate. Fifty microliters of room temperature 2× CPRG (Genlantis, San Diego, CA, USA) was added to each well containing a sample. The plate was read at 570 nm (BioTek, uQuant, Winooski, VT, USA) as soon as any well began to show color change.

## 3. Results

### 3.1. Design of Nested CV-N

**Figure 1:**
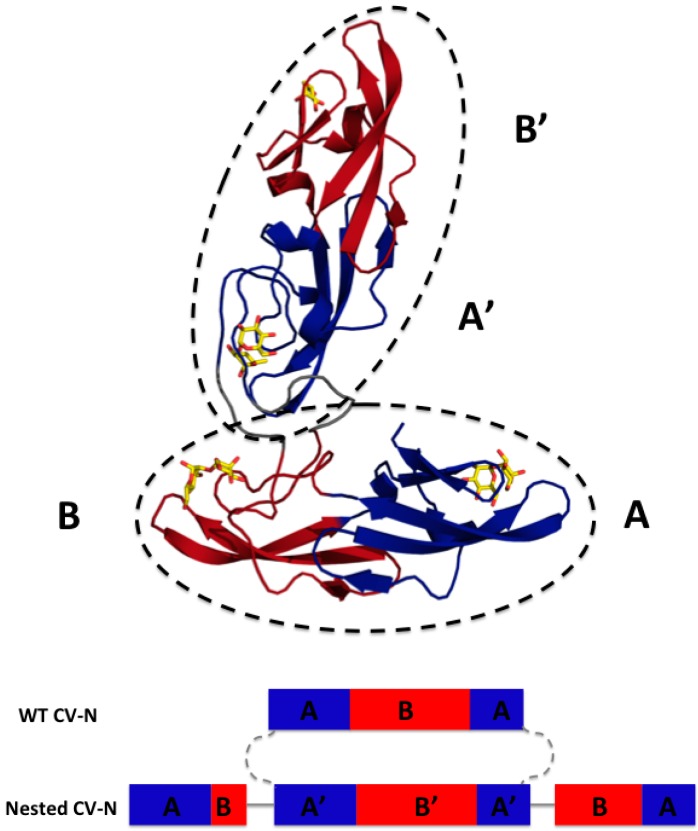
Model of Nested Cyanovirin-N (CV-N): Top panel, glycan binding domains A and A’ are in blue and B and B’ in red; docked dimannose is represented in yellow. The two CV-N folds are enclosed in ellipsoids to highlight the structures (dashed lines). Bottom panel, Sequence arrangement of WT CV-N and designed Nested CV-N construct; as in WT CV-N, the binding domains are not contiguous in the sequence. The sequence of one CV-N protein is spliced into the sequence of a second one and connected by flexible linkers.

**Figure A1:**
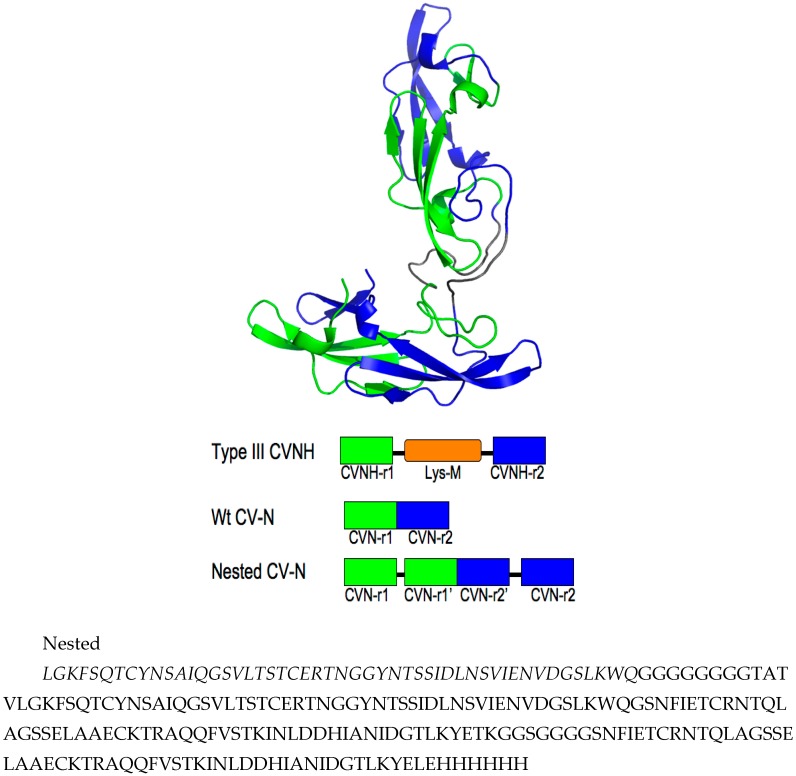
Model of Nested CV-N showing the sequence repeats in the protein. Top panel, sequence repeats 1–50 and 59–109 in green, and 112–162 and 173–222 in blue; note that the sequence repeats do not correspond to the glycan binding domains because binding domains are not contiguous in sequence (see text). Middle panel, Sequence arrangement of Type III CV-NH proteins, WT CV-N, and cloned Nested CV-N construct (adapted from ref. [49]). Bottom panel, sequence of Nested CV-N.

We designed Nested CV-N after Type III CV-NH family members in which the two glycan binding sites of cyanovirin are separated by the insertion of a distinct protein domain (LysM). This suggested that it might be possible to increase ligand multivalency by inserting a second cyanovirin sequence (101 amino acids) in the linker region between the two glycan binding sites (Figure 1). The sequence of Nested CV-N thus starts with amino acids 1–50, which comprise part of site A (1–39) and site B (40–50), followed by a linker (G_8_), and residues 59–162, which comprise a complete CV-N structure (sites A’ and B’), a second linker (GGSGGGGS), and finally residues 173–222, which complete the folds of site B (173–210) and site A (211–222). The full sequence is reported in Supplemental Information (Appendix A) (Figure A1). Based on the structure of a Type III CV-NH protein [40], two cyanovirin domains can be viewed as ellipsoids that are predicted to be nearly orthogonal to each other, creating a tight control of the spatial arrangement of each domain (Figure 1A). Nested CV-N contains two copies of domain A (A, A’) and two copies of domain B (B, B’); as in WT CV-N, the domains are not contiguous in the sequence (Figure 1B). The inserted sequence of CV-N contains a P51G mutation, thus preventing possible formation of domain-swapped dimers. Compared with the rigid structure of the domain-swapped dimers, the flexible linkers connecting the two CV-N units should permit an increased flexibility of the binding domains that we hypothesized might affect engagement with glycosylated gp120. Using the NMR structure of WT CV-N bound to two dimannoses (PDB entry 1IIY) [41] as a template, we docked dimannose in each of the four glycan-binding domains of Nested CV-N (Figure 1). According to our model, the distances between the four binding sites on Nested CV-N range between approximately 15 Å (B-B’) and 45 Å (A-A’), with A-B distances approximately 30 Å; for comparison, distances in the 3D domain-swapped dimer of WT CV-N are in the 30–35 Å range [22,42]. Thus, the Nested CV-N construct should be able to accommodate a broader array of glycan presentation on the surface of gp120.

### 3.2. Nested CV-N Adopts a Native-Like Fold

**Figure 2:**
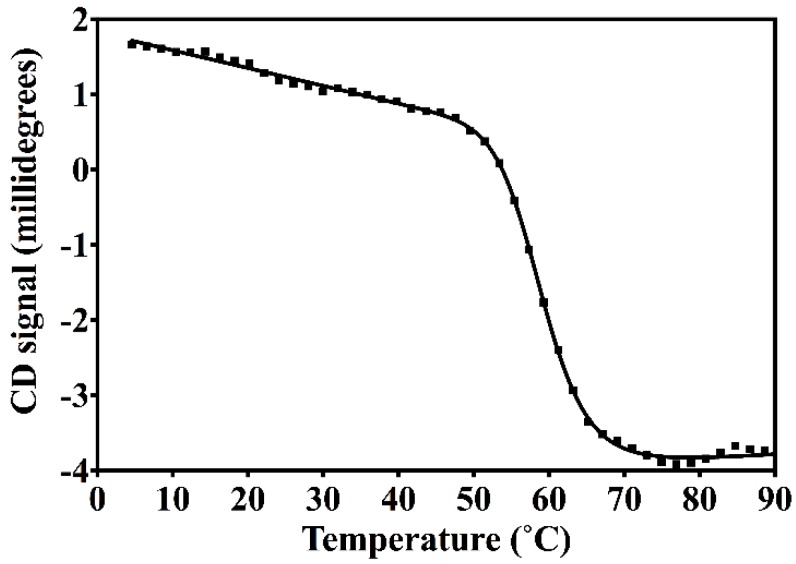
Thermal denaturation profile of Nested CV-N, showing the Circular Dichroism (CD) signal monitored at 235 nm at increasing temperatures in the 4 to 94 °C range for a sample containing 15 µM of protein in 10 mM HEPES, pH = 8.0 buffer. The apparent *T*_m_ was obtained by analyzing the data as described in Methods.

**Figure A2:**
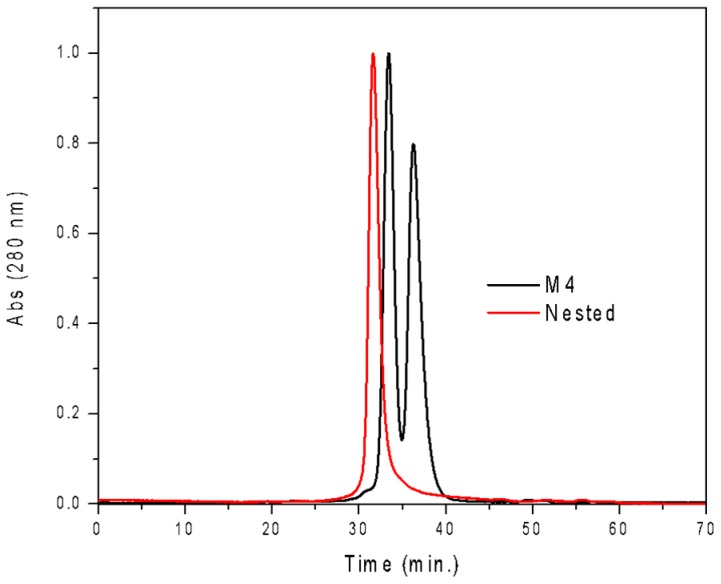
Gel filtration profile of Nested CV-N, showing a single species. CVNm4, which exists as a mixture of monomer and dimer in solution, is shown as comparison.

**Figure A3:**
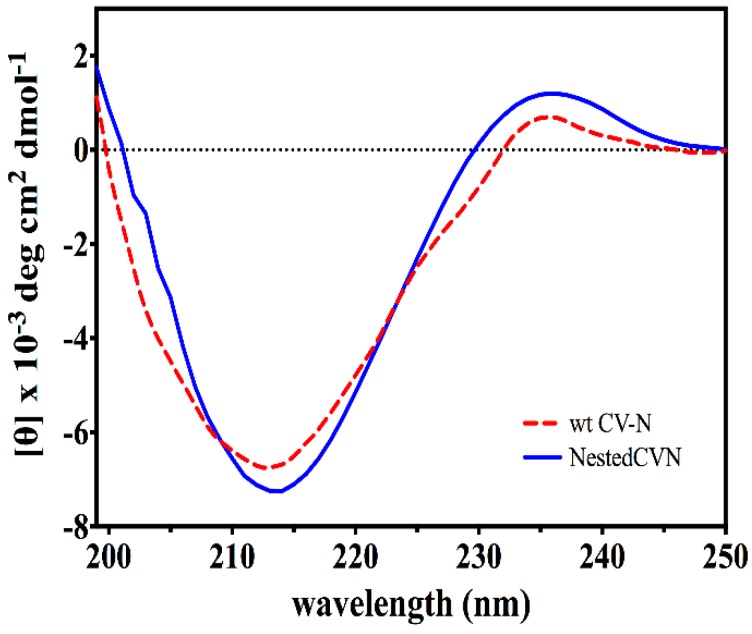
CD spectra of WT CV-N (red) and Nested CV-N (blue). Protein concentration was in the 1–15 µM range, 0.1 M phosphate buffer pH 7.5.

**Figure A4:**
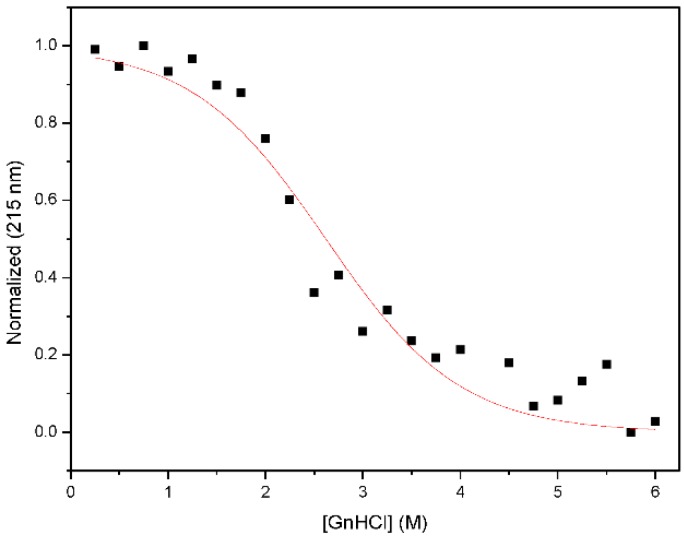
Chemical denaturation profile of Nested CV-N. Samples containing 15 M protein in 0.1 M phosphate buffer, pH 7.4 were prepared at the desired concentration of guanidinium hydrochloride and equilibrated at room temperature for 72 h previous to measurements.

The resulting construct was expressed in *E. coli* and readily purified to homogeneity. Nested CV-N is a monomer as assessed by gel filtration chromatography: the elution profile contains a single peak with retention times comparable to that of the domain-swapped dimer of WT CV-N as expected from the design; the profile displays no evidence of aggregation supporting the use of the P51G substitution to prevent oligomerization (Figure A2). To determine whether Nested CV-N was well folded, we evaluated circular dichroism (CD) and NMR data. The CD spectrum of Nested CV-N displays a minimum at approximately 212 nm and a maximum around 192 nm (Figure A3), features consistent with the presence of mainly β-sheet structure, and closely resembles that of WT CV-N and of P51G-m4-CV-N, indicating that the overall backbone CV-N fold is conserved in Nested CV-N [19,43]. We assessed the thermodynamic stability of Nested CV-N to chemical and thermal denaturation by monitoring the CD signal as a function of either [Gdn·HCl] or temperature. A rigorous analysis of folding parameters obtained from thermal or chemical denaturation is not possible beyond apparent values, since thermal denaturation of CV-N and its mutants is not fully reversible, and chemical denaturation proceeds through at least one folding intermediate [29]. However, direct comparison of the apparent midpoints of the transitions with WT CV-N reveals comparable stability. The thermal denaturation profile of Nested CV-N monitored at a wavelength of 235 nm (Figure 2) shows an apparent T_m_ of 59.0 °C, identical to that of WT CV-N measured in similar conditions [21]. Chemical denaturation profiles were obtained by equilibrating samples containing identical concentration of protein and [Gdn·HCl] in the 0-6 M range at room temperature for 72 h prior to CD readout at 215 mn [29]. The apparent midpoint of the transition *C*_m_ is 2.4 M for Nested CV-N, identical to that of WT CV-N (Figure A4).

**Figure 3:**
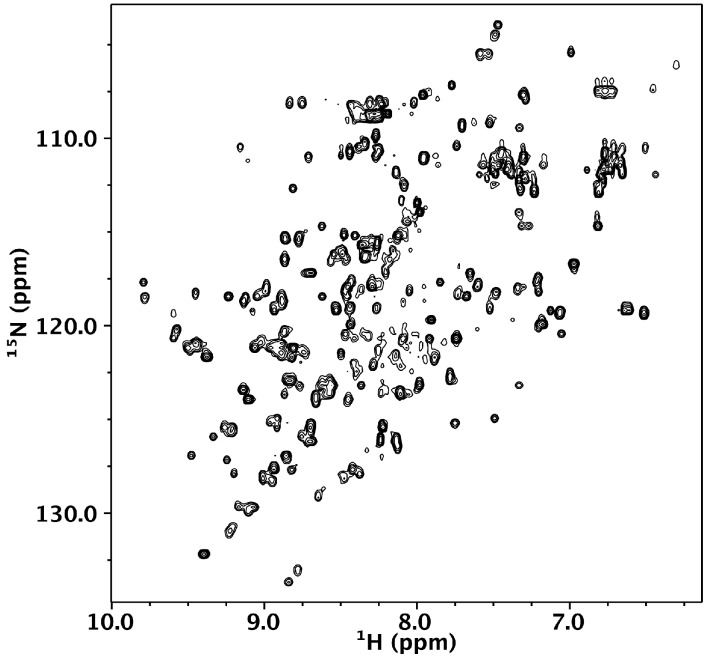
^1^H-^15^N HSQC of 150 µM Nested CV-N in 0.1 M citrate buffer, pH 4.74. The spectrum was collected at 600 MHz at 40 °C.

We turned to NMR spectroscopy to provide site-specific information on whether Nested CV-N is folded throughout its sequence. The ^1^H-^15^N HSQC spectrum gives rise to excellent chemical shift dispersion in both dimensions, indicating the absence of local unfolded structure (Figure 3). Nested CV-N comprises 230 residues, which would typically give rise to over 230 crosspeaks in the HSQC spectrum if its tertiary structure consisted of a single domain. In contrast, we observe ~167 crosspeaks (excluding sidechain resonances), which might arise from either intermediate exchange or from a subset of residues existing in magnetically equivalent environments giving rise to a single crosspeak. This latter idea is consistent with the design of Nested CV-N, which comprises two structurally equivalent CV-N repeats (Figure 1). To evaluate this possibility, we compared wild type CV-N and Nested CV-N spectra and found some residues appear to give rise to a single crosspeak, whereas other residues appear to give rise to two or more crosspeaks with similar chemical shifts. We interpret these data to reflect that some residues in Nested CV-N adopt a unique chemical environment identical to wild type CV-N, whereas other residues adopt slightly different chemical environments resulting in crosspeak doubling. In some cases the linewidths from these crosspeak pairs differed significantly enough to suggest different protein dynamics between the Nested domains. To further evaluate this possibility, we tentatively assigned 40% of the Nested CV-N spectrum from visual inspection of the monomeric wild type CV-N spectrum. Chemical shift assignments from 44 out of 110 wild type CV-N residues could be transferred to the Nested spectrum with reasonable confidence. Of these 44 residues, 10 resulted in two crosspeaks for each residue (T7, Q14, L48, N30, L36, D44, W50, A70, G96, T97) including residues involved in both the high and low affinity binding sites. Thus, no correlation between structure and crosspeak doubling was apparent eliminating the possibility that the observed conformational heterogeneity derived from a specific interface. We conclude that the Nested CV-N construct adopts the intended fold albeit with slight differences in structure and dynamics between the two CV-N repeats.

### 3.3. Binding to Glycans and Antiviral Activity

**Figure 4:**
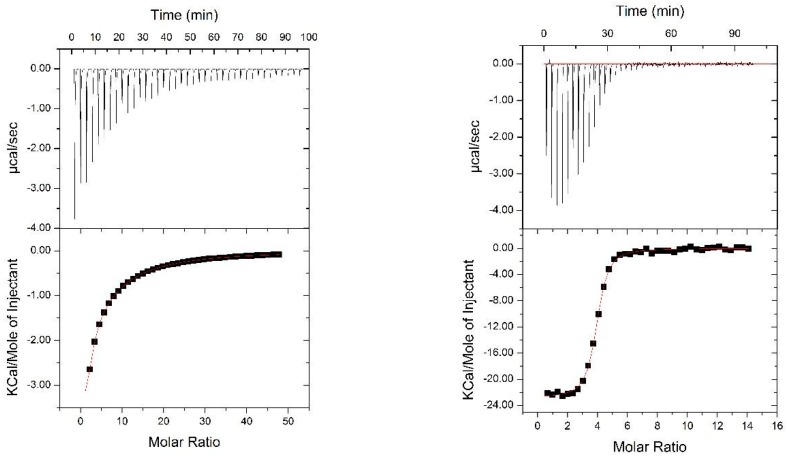
ITC titrations of Nested CVN with dimannose (left panel ) and with Man9 (right panel).

We evaluated the functional integrity of the glycan-binding domains comprised within Nested CV-N by analyzing its ability to bind dimannose, as well as complex oligomannosides (Man-5 through Man-9) displayed on protein surfaces. The structures of the complex Man-9 and Man-8 glycans contain Man(1 → 2)Man termini, one of which is also part of a linear trimannose (the D3, D2, and D1 arms), and thus can satisfy the binding requirements of domains A and B; in Man-8, the central D2 arm is missing one mannose unit; smaller oligomannosides contain Man (1 → 2)Man termini [44]. To this end, we first assessed binding of Nested CV-N to dimannose (Man (1 → 2)Man and to Man9 in solution by isothermal titration calorimetry (ITC). The data are shown in Figure 4. Titration with dimannose revealed the presence of two sets of binding sites, one with high affinity and one with low affinity, each with a stoichiometry of two. Analyses of the binding events yields *K*_d_ = 25 ± 2 mM and *K*_d_ = 950 ± 8 µM, respectively (see Methods for details). The dissociation constants for the two sites are comparable to those obtained for WT CV-N, which also contains a high-affinity binding site (*K*_d_ = 15.3 µM) and a low-affinity one (*K*_d_ = 400 µM). These results are consistent with NMR-monitored titrations by which the high affinity site was assigned to domain B, and the lower affinity site to domain A [11,41]. In contrast, binding to Man-9 involves four sites, each with *K*_d_ = 1.10 ± 0.07 µM; the four sites appear indistinguishable, possibly because the differences in binding affinities within the two type of domains are too small for detection, or because the presence of both dimannose and linear trimannose in the Man-9 structure satisfies the intrinsic glycan preference of each domain. This value is comparable with the dissociation constant of 0.488 ± 0.071 µM reported for 1:1 binding of WT CV-N to Man-8 [20].

**Figure 5:**
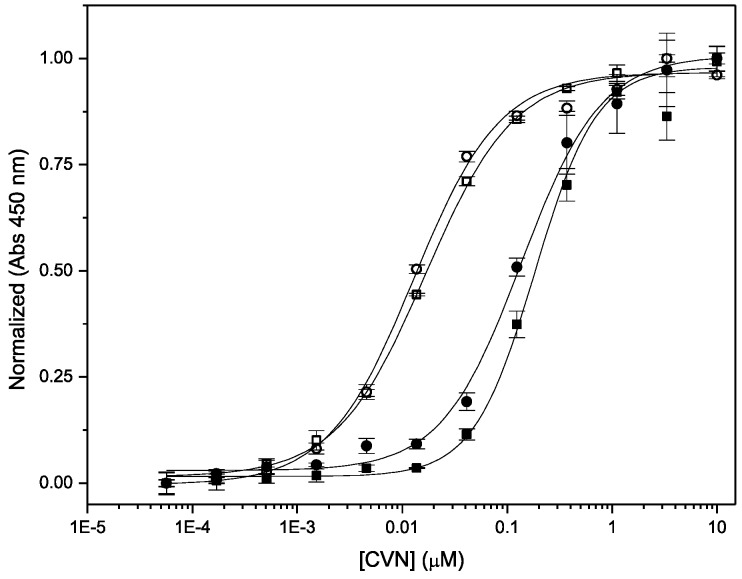
ELISA traces for binding of Nested CV-N (circles) and WT CV-N (squares) to gp120 (black) and RNase B (filled symbols).

Binding of Nested CV-N to complex oligomannosides on protein surfaces was assessed using ELISA on immobilized gp120 and ribonuclease B (Figure 5). Ribonuclease B contains a single N-linked glycosylation site, populated with a mixture of Man-5, Man-6, Man-7, Man-8 and Man-9 isoforms [45,46]. Previously, we have shown that WT CV-N recognizes Man-8 and Man-9 isoforms on the surface of ribonuclease B, but not the other smaller isoforms. ITC measurements revealed that binding to Man-8 with a 1:1 stoichiometry and a sub-micromolar dissociation constant [34]. The assays were conducted in parallel with WT CV-N as internal control; to avoid possible differences in affinity with available anti-CVN antibodies, the proteins were detected with HRP-conjugated His-tag specific reagents as described in Methods. We found that Nested CV-N interacts with both glycoproteins with apparent affinity comparable to WT CV-N. The EC_50_ values for binding to gp120 are 1.29 ± 0.11 × 10^−9^ M for Nested CV-N and 1.66 ± 0.35 × 10^−9^ M for WT CV-N. Testing with ribonuclease B resulted in very similar EC_50_ values of 1.3 ± 0.1 × 10^−8^ M and 1.7 ± 0.2 × 10^−8^ M respectively for Nested CV-N and for WT CV-N. As observed before, binding to ribonuclease B is slightly weaker compared to binding to gp120, because only a portion of the ribonuclease B bound to the ELISA plate actually bears either Man-8 or Man-9. Furthermore, ribonuclease B contains a single glycosylation site for CV-N binding, whereas gp120 contains about 25 sites per monomer [34,44].

Having assessed the ability of Nested CV-N to bind glycans with high affinity, we evaluated its anti-HIV activity using a modified XTT-tetrazolium assay. This assay measures cell viability when exposed to infective HIV virions in the presence of increasing concentrations of added inhibitor, over a period of one week at 38 °C [36]. Nested CV-N was compared to WT CV-N from two source laboratories and to P51G-CVN, which contains the same hinge region mutations present in the central CV-N repeat in Nested CV-N. The P51G mutation stabilizes the monomeric version of CV-N, and disfavors the formation of the 3D domain-swapped form by increasing the energy barrier for the interconversion between the two forms. We found that the activity of Nested CV-N is slightly increased compared to that of WT CV-N, showing 2-5 fold improvement compared to WT CV-N, and approximately 8 times compared to P51G-CVN; all proteins are active at sub-nanomolar concentrations. The EC_50_ values are shown in Table 1. We further confirmed these data by assessing the ability of Nested CV-N to block viral entry into target cells. This assay evaluates the inhibition of HIV-mediated cell fusion, using a vaccinia virus-based gene reporter system adapted from the procedure developed by Berger and colleagues [37]. We found that Nested CV-N exhibited inhibitory activity similar to WT CV-N, as shown by IC_50_ values of 7.5 ± 0.6 nM and 18 ± 0.5 nM, respectively (Table 1).

These data provide further support to a correlation between number of functional glycan-binding domains and antiviral activity for CV-N. P51G-CVN, which is found exclusively as a monomer upon incubation at 38 °C, should present only two binding domains in the conditions of the assay, and it is the least active of the series. Nested CV-N, which contains four functional glycan binding modules as confirmed by ITC, shows the highest activity. WT CV-N, which can access both the monomeric and the 3D domain swapped form, shows intermediate activity.

## 4. Conclusions

We adopted the domain architecture of Type III CV-N homologous family (CV-NH) proteins to design Nested CV-N, predicted to contain two independently folded CV-N structures connected by linkers, providing enhanced multivalency. Nested CV-N appears to adopt the intended fold comprising two near identical WT CV-N repeats. The two-dimensional NMR spectrum demonstrates that many of the Nested residues exhibit identical or near-identical chemical shifts indicating that these residues experience identical chemical environments. Further, Nested CV-N is stable to thermal denaturation, and contains four functional dimannose binding domains, two with high affinity and two with low affinity as assessed by ITC, consistent with the presence of two sets of domains as observed in WT CV-N. The difference in affinity between the two domains is lost when Nested CV-N is assayed with Man9, which comprises both linear dimannose and trimannose and thus can satisfy the glycan recognition specificity of each domain [9,26,47]. Affinity for Man9, which more closely resembles the complex glycans found on protein surfaces, is higher than for dimannose (approx. 1 µM compared with 25 μM and 900 µM for dimannose). The advantage conferred by the presence of multiple glycan-binding domain is also seen in binding to heavily glycosylated proteins: compared to sparsely glycosylated ribonuclease B, Nested CV-N binds gp120 with EC_50_ at 10 times lower concentrations. The activity of Nested CV-N against HIV in cellular assays is comparable to that of WT CV-N: A slight increase of 2–5 fold observed may indicate that all binding domains are engaged simultaneously, and will be further investigated. We note that the activity of Nested CV-N is enhanced 8-fold compared to P51G-CVN, which cannot form domain swapped dimers. The increase in activity observed with Nested CV-N was confirmed in fusion inhibition assays using indicator cells.

Over the years, the relationship between multivalency and antiviral potency in antiviral lectins has been the object of vigorous investigation. Through the use of site directed mutagenesis it was established that at least two intact glycan binding sites are necessary for the antiviral activity of CV-N, but it remained unclear whether the activity could be augmented over that of WT CV-N by augmenting the number of domains [19,21,24,25,27]. Our results confirm the importance of multivalency in the antiviral activity of CV-N, and support the development of flexible covalent dimers as a means to simultaneously engage all four glycan-binding sites. The robustness of the CV-N fold to reengineering of its glycan binding sites[11,29,48] may lead to future designs built on Nested CV-N that further augment its activity and/or modulate its specificity, and which could be used as specific recognition modules for glycans.

## Figures and Tables

**Table 1:** Glycan binding and anti-HIV-1 activity of Nested CV-N and WT CV-N.

Protein	Binding to gp120 EC_50_ (nM)	Antiviral Activity EC_50_ (nM)	Fusion Inhibition IC_50_ (nM)
Nested CV-N	1.29 ± 0.11	0.1 ± 0.02	7.5 ± 0.6
WT CV-N	1.66 ± 0.35	0.2 ± 0.04	18 ± 0.4
WT CV-N (MTL) ^a^	N/A	0.5 ± 0.08	
P51G-CVN	4 ^b^	0.8 ± 0.04	

^a^ A second wild-type CV-N control produced in the Molecular Targets Laboratory. All sample quantities were determined by amino acid analysis. ^b^ From Reference [18].

## References

[1] O’Keefe B.R., Smee D.F., Turpin J.A., Saucedo C.J., Gustafson K.R., Mori T., Blakeslee D., Buckheit R., Boyd M.R. (2003). Antimicrob. Agents Chemother..

[2] Helle F., Wychowski C., Vu-Dac N., Gustafson K.R., Voisset C., Dubuisson J. (2006). Cyanovirin-N inhibits hepatitis C virus entry by binding to envelope protein glycans. J. Biol. Chem..

[3] Balzarini J., Van Laethem K., Hatse S., Froeyen M., Peumans W., Van Damme E., Schols D. (2005). Carbohydrate-binding agents cause deletions of highly conserved glycosylation sites in HIV GP120: A new therapeutic concept to hit the achilles heel of HIV. J. Biol. Chem..

[4] Balzarini J. (2007). Carbohydrate-binding agents: A potential future cornerstone for the chemotherapy of enveloped viruses?. Antivir. Chem. Chemother..

[5] Koharudin L.M., Gronenborn A.M. (2014). Antiviral lectins as potential HIV microbicides. Curr. Opin. Virol..

[6] Bewley C.A., Gustafson K.R., Boyd M.R., Covell D.G., Bax A., Clore G.M., Gronenborn A.M. (1998). Solution structure of cyanovirin-N, a potent HIV-inactivating protein. Nat. Struct. Biol..

[7] Bewley C.A. (2001). Solution structure of a cyanovirin-N:Man alpha 1-2Man alpha complex: Structural basis for high-affinity carbohydrate-mediated binding to gp120. Structure.

[8] Margulis C.J. (2005). Computational study of the dynamics of mannose disaccharides free in solution and bound to the potent anti-HIV virucidal protein cyanovirin. J. Phys. Chem. B.

[9] Fujimoto Y.K., Green D.F. (2012). Carbohydrate recognition by the antiviral lectin cyanovirin-N. J. Am. Chem. Soc..

[10] Barrientos L.G., Matei E., Lasala F., Delgado R., Gronenborn A.M. (2006). Dissecting carbohydrate-Cyanovirin-N binding by structure-guided mutagenesis: Functional implications for viral entry inhibition. Protein Eng. Des. Sel..

[11] Bolia A., Woodrum B.W., Cereda A., Ruben M.A., Wang X., Ozkan S.B., Ghirlanda G. (2014). A flexible docking scheme efficiently captures the energetics of glycan-cyanovirin binding. Biophys. J..

[12] Vorontsov I.I., Miyashita O. (2009). Solution and crystal molecular dynamics simulation study of m4-cyanovirin-N mutants complexed with di-mannose. Biophys. J..

[13] Fujimoto Y.K., Terbush R.N., Patsalo V., Green D.F. (2008). Computational models explain the oligosaccharide specificity of cyanovirin-N. Protein Sci..

[14] Barrientos L.G., Gronenborn A.M. (2002). The domain-swapped dimer of cyanovirin-N contains two sets of oligosaccharide binding sites in solution. Biochem. Biophys. Res. Commun..

[15] Yang F., Bewley C.A., Louis J.M., Gustafson K.R., Boyd M.R., Gronenborn A.M., Clore G.M., Wlodawer A. (1999). Crystal structure of cyanovirin-N, a potent HIV-inactivating protein, shows unexpected domain swapping. J. Mol. Biol..

[16] Botos I., Mori T., Cartner L.K., Boyd M.R., Wlodawer A. (2002). Domain-swapped structure of a mutant of cyanovirin-N. Biochem. Biophys. Res. Commun..

[17] Ziolkowska N.E., O’Keefe B.R., Mori T., Zhu C., Giomarelli B., Vojdani F., Palmer K.E., McMahon J.B., Wlodawer A. (2006). Domain-swapped structure of the potent antiviral protein griffithsin and its mode of carbohydrate binding. Structure.

[18] Barrientos L.G., Lasala F., Delgado R., Sanchez A., Gronenborn A.M. (2004). Flipping the switch from monomeric to dimeric CV-N has little effect on antiviral activity. Structure.

[19] Fromme R., Katiliene Z., Giomarelli B., Bogani F., Mahon J.M., Mori T., Fromme P., Ghirlanda G. (2007). A Monovalent Mutant of Cyanovirin-N Provides Insight into the Role of Multiple Interactions with gp120 for Antiviral Activity. Biochemistry.

[20] Shenoy S.R., Barrientos L.G., Ratner D.M., O’Keefe B.R., Seeberger P.H., Gronenborn A.M., Boyd M.R. (2002). Multisite and multivalent binding between cyanovirin-N and branched oligomannosides: Calorimetric and NMR characterization. Chem. Biol..

[21] Liu Y., Carroll J.R., Holt L.A., McMahon J., Giomarelli B., Ghirlanda G. (2009). Multivalent interactions with gp120 are required for the anti-HIV activity of Cyanovirin. Biopolymers.

[22] Keeffe J.R., Gnanapragasam P.N., Gillespie S.K., Yong J., Bjorkman P.J., Mayo S.L. (2011). Designed oligomers of cyanovirin-N show enhanced HIV neutralization. Proc. Natl. Acad. Sci. USA.

[23] Koharudin L.M.I., Gronenborn A.M. (2012). Sweet entanglements-protein: Glycan interactions in two HIV-inactivating lectin families. Biopolymers.

[24] Matei E., Furey W., Gronenborn A.M. (2008). Solution and crystal structures of a sugar binding site mutant of cyanovirin-N: No evidence of domain swapping. Structure.

[25] Matei E., Zheng A., Furey W., Rose J., Aiken C., Gronenborn A.M. (2010). Anti-HIV activity of defective cyanovirin-N mutants is restored by dimerization. J. Biol. Chem..

[26] Woodrum B.W., Maxwell J.D., Bolia A., Ozkan S.B., Ghirlanda G. (2013). The antiviral lectin cyanovirin-N: Probing multivalency and glycan recognition through experimental and computational approaches. Biochem. Soc. Trans..

[27] Fromme R., Katiliene Z., Fromme P., Ghirlanda G. (2008). Conformational gating of dimannose binding to the antiviral protein cyanovirin revealed from the crystal structure at 1.35 A resolution. Protein Sci..

[28] Greenfield N.J. (2006). Using circular dichroism spectra to estimate protein secondary structure. Nat. Protoc..

[29] Patsalo V., Raleigh D.P., Green D.F. (2011). Rational and computational design of stabilized variants of cyanovirin-N that retain affinity and specificity for glycan ligands. Biochemistry.

[30] Delaglio F., Grzesiek S., Vuister G.W., Zhu G., Pfeifer J., Bax A. (1995). NMRPipe: A multidimensional spectral processing system based on UNIX pipes. J. Biomol. NMR.

[31] Johnson B.A., Blevins R.A. (1994). NMR View: A computer program for the visualization and analysis of NMR data. J. Biomol. NMR.

[32] Johnson B.A. (2004). Using NMRView to visualize and analyze the NMR spectra of macromolecules. Methods Mol. Biol..

[33] Boyd M.R., Gustafson K.R., McMahon J.B., Shoemaker R.H., O’Keefe B.R., Mori T., Gulakowski R.J., Wu L., Rivera M.I., Laurencot C.M. (1997). Discovery of cyanovirin-N, a novel human immunodeficiency virus-inactivating protein that binds viral surface envelope glycoprotein gp120: Potential applications to microbicide development. Antimicrob. Agents Chemother..

[34] Shenoy S.R., O’Keefe B.R., Bolmstedt A.J., Cartner L.K., Boyd M.R. (2001). Selective interactions of the human immunodeficiency virus-inactivating protein cyanovirin-N with high-mannose oligosaccharides on gp120 and other glycoproteins. J. Pharmacol. Exp. Ther..

[35] Gulakowski R.J., McMahon J.B., Staley P.G., Moran R.A., Boyd M.R. (1991). A semiautomated multiparameter approach for anti-HIV drug screening. J. Virol. Methods.

[36] Nussbaum O., Broder C.C., Berger E.A. (1994). Fusogenic mechanisms of enveloped-virus glycoproteins analyzed by a novel recombinant vaccinia virus-based assay quantitating cell fusion-dependent reporter gene activation. J. Virol..

[37] Alkhatib G., Broder C.C., Berger E.A. (1996). Cell type-specific fusion cofactors determine human immunodeficiency virus type 1 tropism for T-cell lines *versus* primary macrophages. J. Virol..

[38] Fuerst T.R., Niles E.G., Studier F.W., Moss B. (1986). Eukaryotic transient-expression system based on recombinant vaccinia virus that synthesizes bacteriophage T7 RNA polymerase. Proc. Natl. Acad. Sci. USA.

[39] Broder C.C., Berger E.A. (1995). Fusogenic selectivity of the envelope glycoprotein is a major determinant of human immunodeficiency virus type 1 tropism for CD4+ T-cell lines *vs.* primary macrophages. Proc. Natl. Acad. Sci. USA.

[40] Koharudin L.M.I., Viscomi A.R., Montanini B., Kershaw M.J., Talbot N.J., Ottonello S., Gronenborn A.M. (2011). Structure-Function Analysis of a CVNH-LysM Lectin Expressed during Plant Infection by the Rice Blast Fungus Magnaporthe oryzae. Structure.

[41] Bewley C.A., Otero-Quintero S. (2001). The Potent Anti-HIV Protein Cyanovirin-N Contains Two Novel Carbohydrate Binding Sites That Selectively Bind to Man 8D1D3 and Man 9with Nanomolar Affinity: Implications for Binding to the HIV Envelope Protein gp120. J. Am. Chem. Soc..

[42] Barrientos L.G., Louis J.M., Botos I., Mori T., Han Z., O’Keefe B.R., Boyd M.R., Wlodawer A., Gronenborn A.M. (2002). The domain-swapped dimer of cyanovirin-N is in a metastable folded state: Reconciliation of X-ray and NMR structures. Structure.

[43] Barrientos L.G., Louis J.M., Hung J., Smith T.H., O’Keefe B.R., Gardella R.S., Mori T., Boyd M.R., Gronenborn A.M. (2002). Design and initial characterization of a circular permuted variant of the potent HIV-inactivating protein cyanovirin-N. Proteins.

[44] Scanlan C.N., Offer J., Zitzmann N., Dwek R.A. (2007). Exploiting the defensive sugars of HIV-1 for drug and vaccine design. Nature.

[45] Prien J.M., Ashline D.J., Lapadula A.J., Zhang H. (2009). The high mannose glycans from bovine ribonuclease B isomer characterization by ion trap MS. J. Am. Soc. Mass Spectrom..

[46] Kawasaki N., Ohta M., Hyuga S., Hashimoto O., Hayakawa T. (1999). Analysis of carbohydrate heterogeneity in a glycoprotein using liquid chromatography/mass spectrometry and liquid chromatography with tandem mass spectrometry. Anal. Biochem..

[47] Sandstrom C., Berteau O., Gemma E., Oscarson S., Kenne L., Gronenborn A.M. (2004). Atomic mapping of the interactions between the antiviral agent cyanovirin-N and oligomannosides by saturation-transfer difference NMR. Biochemistry.

[48] Koharudin L.M.I., Furey W., Gronenborn A.M. (2009). A designed chimeric cyanovirin-N homolog lectin: Structure and molecular basis of sucrose binding. Proteins.

[49] Percudani R., Montanini B., Ottonello S. (2005). The anti-HIV cyanovirin-N domain is evolutionarily conserved and occurs as a protein module in eukaryotes. Proteins.

